# A versatile chemical toolbox drives plant regeneration

**DOI:** 10.1093/hr/uhag155

**Published:** 2026-04-17

**Authors:** Yin Song, Langrang Zhang, Ziyao Hu, Varvara E Tvorogova, Ludmila A Lutova, Huibin Han, Guodong Wang

**Affiliations:** State Key Laboratory for Crop Stress Resistance and High-Efficiency Production, College of Agronomy, Northwest A&F University, Yangling 712100, China; College of Life Sciences, Shaanxi Normal University, Xi’an 710119, China; College of Life Sciences, Shaanxi Normal University, Xi’an 710119, China; Department of Genetics and Biotechnology, Saint Petersburg State University, 7/9 Universitetskaya Embankment, Saint Petersburg 199034, Russia; Plant Biology and Biotechnology Department, Sirius University of Science and Technology, 1 Olympic Avenue, Sochi 354340, Russia; Department of Genetics and Biotechnology, Saint Petersburg State University, 7/9 Universitetskaya Embankment, Saint Petersburg 199034, Russia; Plant Biology and Biotechnology Department, Sirius University of Science and Technology, 1 Olympic Avenue, Sochi 354340, Russia; Jiangxi Province Key Laboratory of Vegetable Cultivation and Utilization; Research Center of Plant Functional Genes and Tissue Culture Technology, College of Bioscience and Bioengineering, Jiangxi Agricultural University, Nanchang 330045, China; College of Life Sciences, Shaanxi Normal University, Xi’an 710119, China

## Abstract

Plant regeneration is widely exploited in genetic transformation, genome editing, tissue culture, cuttage, and grafting, which are crucial means to improve crop productivity and resilience. However, plant regeneration is heavily restricted by their low regeneration efficiency and genotype recalcitrance, especially in many economically important plant species. To address these challenges, new approaches have been developed to greatly improve regeneration efficiency and genotype plasticity. Among them, small molecules represent attractive options that offer an effective, inexpensive, and convenient strategy to enhance regeneration efficiency in a stepwise and controllable manner. Here, we summarize current advances in the characterization of small molecules with superior efficacy and their molecular mechanisms in plant regeneration, highlighting their advantages for fundamental studies and practical applications. Finally, we conclude with a critical discussion on the remaining questions and future challenges in pinpointing small molecules with regeneration-enhancing potential.

## Introduction

Plant regeneration underpins key techniques in genetic engineering and plant biotechnology such as genetic transformation, genome editing, tissue culture, wound healing, cuttage, and grafting, which are essential for crop enhancements [1–3]. While plants possess a remarkable capacity for regeneration, this potential is highly species- and genotype-dependent, making low plant regeneration efficiency a major bottleneck in crop enhancement using genetic breeding and plant biotechnology [3, 4]. Substantial efforts, therefore, have focused on developing new strategies to enhance regeneration capacity. For instance, ectopic expression of developmental regulators such as *WUSCHEL* (*WUS*) and *BABY BOOM* (*BBM*) has been shown to dramatically improve the efficiency of embryogenesis or organogenesis from somatic cells in various crops [1, 4–6]. However, their application remains constrained because it still requires lengthy, laborious, and costly tissue culture and subsequent genetic transformation to generate the component genetic materials. Furthermore, forced expression of such developmental regulators often leads to undesirable pleiotropic effects, including developmental abnormalities and reduced fertility [1, 4]. In addition, achieving precise temporal and tunable control over such regulators presents considerable technical complexities and challenges, typically requiring ectopic expression of developmental regulators and timely excision of introduced genetic components [1, 3, 4].

In 1957, Skoog and Miller demonstrated that during *in vitro* culture, the balance between exogenous auxin and cytokinin could dramatically direct organ formation and tissue regeneration [7], already pinpointing the profound role of chemicals in controlling plant regeneration. It is well documented that synthetic chemical analogs to auxin, such as 2,4-dichlorophenoxyacetic, or 1-naphthaleneacetic acid, are routinely used to promote callus induction in plant tissue culture [8]. In principle, *in vitro* plant organogenesis and embryogenesis are primarily built upon chemical reprogramming through the coaction of two elementary chemicals, auxin and cytokinin. As such, chemical-induced *in vitro* regeneration is the most commonly used approach for *de novo* organogenesis in various plant species for propagation, genetic transformation, generation of virus-free plants, and genome editing [9]. Likewise, the chemical strategies parallel advances in regenerative medicine, where small molecules are used to chemically reprogram cell fate by precisely modulating epigenetic states and/or pluripotency factors, thereby providing powerful approaches to generate human pluripotent stem cells [10–12]. These findings together illuminate that somatic cells of both mammals and plants can be chemically reprogrammed to regenerate through *in vitro* culture using proper chemicals, emphasizing the power of chemical-mediated regeneration.

A growing body of evidence in plants highlights the potential of more small molecules in optimizing the tissue culture media for successful and improved plant regeneration. Without genetic engineering, these small molecules have offered distinct advantages for enhancing regeneration capacity, such as convenient, rapid, highly controllable, precisely regulating of dose and treatment duration, and bypassing compensatory biological feedbacks that often occur with genetic manipulations. By mimicking natural developmental cues, the application of such small molecules provides a controllable, genotype-independent strategy to overcome regeneration bottlenecks and advance crop improvement, therefore leading to improved crop productivity and resilience. Here, we summarize current advances in the identification of small molecules with superior efficacy for plant regeneration, highlighting their advantages for both fundamental studies and practical applications. We conclude with a critical discussion of the key challenges in pinpointing small molecules with regeneration-enhancing potential.

### Small molecules facilitate somatic embryogenesis

**Figure 1:**
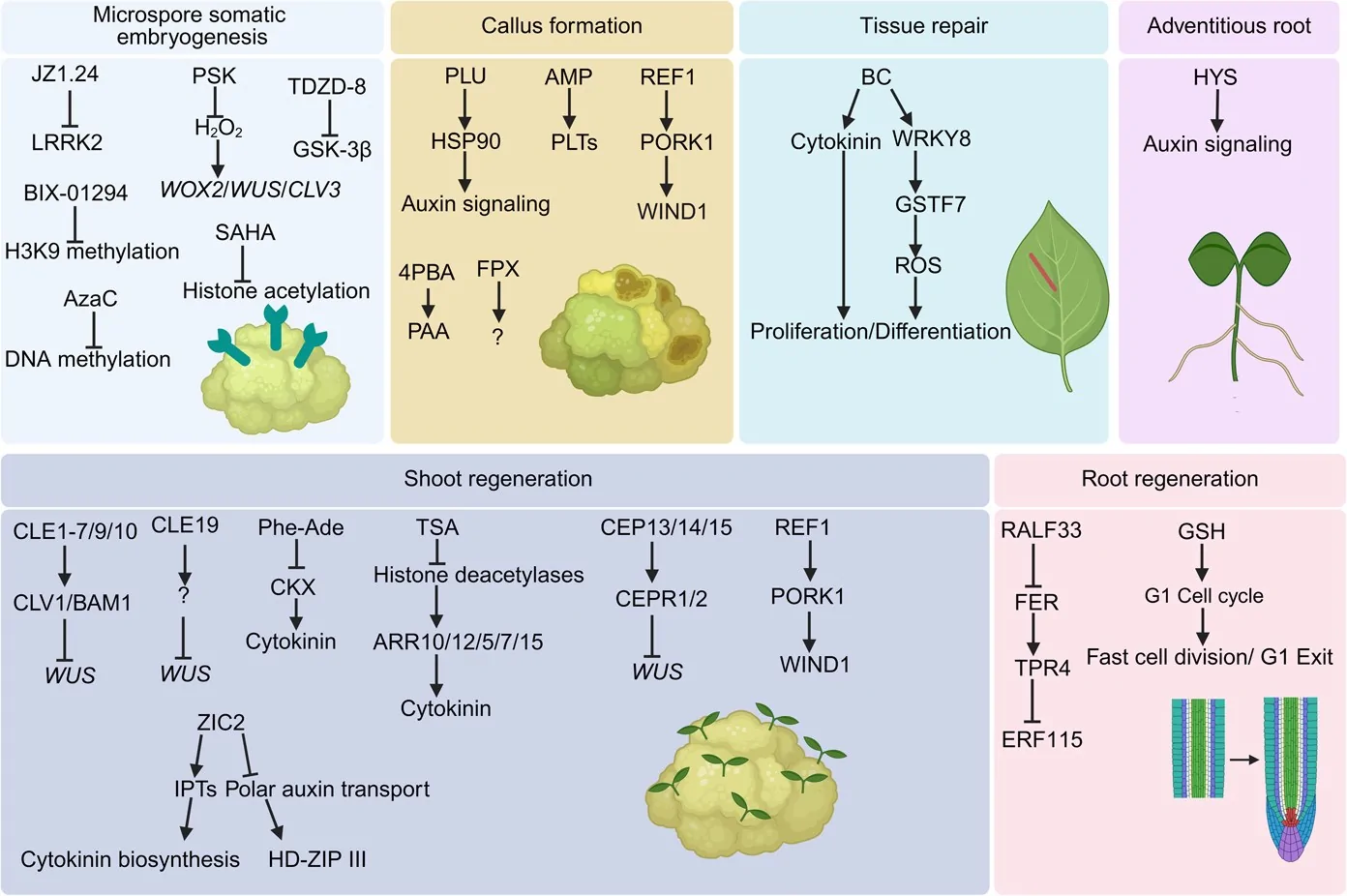
Small molecules regulate plant regeneration capacity. Representative small molecules and small signaling peptides involved in plant regeneration processes, including callus induction, *de novo* shoot and root organogenesis, somatic embryogenesis, and adventitious root formation. These small molecules (de)activate distinct regulatory mechanisms such as epigenetic regulation, plant hormone signaling, or key pluripotency factors to modulate plant regeneration capacity.

Somatic embryogenesis is widely exploited for plant regeneration and genetic transformation across diverse plant species. Epigenetic modification inhibitors, such as 5-Azacytidine (AzaC) [13], BIX-01294 [14], and suberoylanilide hydroxamic acid (SAHA) [15], have been shown to enhance microspore somatic embryogenesis through distinct mechanisms (Fig. 1). Exogenous application of a low concentration of the DNA methylation inhibitor AzaC significantly promotes microspore somatic embryogenesis, while higher concentrations of AzaC inhibit this process [13]. Further investigations indicate that the short-term induction of somatic embryogenesis by AzaC correlates with reduced global DNA methylation, altered 5-methyl-deoxycytidine (5mdC) levels, and changes in chromatin condensation. Similarly, brief exposure to BIX-01294 (diazepin-quinazolin-amine derivative), an H3K9 methylation inhibitor, stimulates somatic embryogenesis [14]. Furthermore, microspore cultures treated with SAHA, a selective inhibitor of class I and II histone deacetylases (HDACs), significantly enhance proembryo production by upregulating the expression of key embryogenic transcription factors such as *FUSCA3* (*FUS3*), *LEAFY COTYLEDON2* (*LEC2*), and *AGAMOUSLIKE15* (*AGL15*), thereby facilitating increased microspore reprogramming rates [15]. Collectively, these findings highlight the potential of epigenetic modification inhibitors as effective tools for improving plant regeneration efficiency.

TDZD-8 (small heterocyclic thiadiazolidinone), a mammalian glycogen synthase kinase 3β inhibitor, also promotes somatic embryogenesis in plants by activating brassinosteroid (BR) signaling (Fig. 1; [16]). *In vitro* application of TDZD-8 to microspore cultures results in improved embryogenesis induction efficiency. Consistent with its promotive role in embryogenesis, TDZD-8 treatment, similar to SAHA, can significantly elevate transcriptional levels of *FUS3*, *LEC2*, and *AGL15* [16]. Further analysis reveals that TDZD-8 activates BR signaling by modulating the transcription of genes involved in BR biosynthesis, catabolism, and signaling. Notably, TDZD-8 appears to function in a genotype-independent manner, as it improves microspore embryogenesis in diverse species including *Brassica napus*, *Hordeum vulgare*, and *Quercus suber* [16].

In addition, small-molecule inhibitors targeting the mammalian LEUCINE-RICH REPEAT KINASE 2, including benzothiazole derivatives JZ1.24, JZ1.3, and JZ1.6, and the indolinone derivative IGS4.75, have also been shown to improve the induction efficiency of microspore somatic embryogenesis [17]. The representative compound JZ1.24 upregulates the expression of BR-related genes such as *BIN2*, *CPD*, *BAS1*, and *SERK1-like*, thereby activating BR signaling and promoting somatic embryogenesis (Fig. 1).

### Chemical induction of hypocotyl adventitious root formation

In response to wounding signals, plant hypocotyls initiate the formation of adventitious roots (ARs) through complex regulatory networks [18, 19]. The small molecule HYSPARIN (HYS) has been demonstrated to facilitate AR development in hypocotyls (Fig. 1; [20]). Application of HYS significantly enhances AR formation in Arabidopsis, rice, and tomato without affecting primary root growth. Notably, HYS fails to induce AR formation in key auxin signaling mutants such as *tmks*, *tir1*, and *arf7 arf19* [20], the corresponding genes of which are critical components of auxin signaling [21]. Transcriptomic analyses further reveal key downstream components such as SAUR19, OFP4, and AGC2, which are involved in the HYS-mediated AR induction. Collectively, these findings indicate that HYS functions through the canonical and cell-surface auxin signaling pathways, effectively mimicking auxin agonist activity to stimulate AR formation [20].

### Small chemicals modulate callus formation and *de novo* organogenesis

Plant tissue culture involves callus induction, a process influenced by several small molecules, including fipexide (FPX, 1-[2-(4-chlorophenoxy)-acetyl]-4-(3,4-ethylenedioxobenzyl)-4-piperazine) [22], PLU (2-furancarboxylic acid, 3-methyl-, 2-[[3-(aminocarbonyl)-2-thienyl]amino]-2-oxoethyl ester) [23], and adenosine monophosphate (AMP) [24]. Initially identified as an inhibitor of hypocotyl elongation, prolonged FPX treatment results in callus induction and formation in the apical meristem regions of Arabidopsis shoots and roots (Fig. 1; [22]). Importantly, FPX demonstrates a conserved function in promoting callus formation and shoot regeneration across various species, including rice, *Populus*, *Brachypodium distachyon*, cucumber, tomato, and soybean, highlighting its broad applicability across different plant species. Transcriptomic analyses have pinpointed FPX-responsive genes, whereas the precise mechanisms underlying FPX-mediated callus induction and shoot regeneration remain to be elucidated.

The PLURIPOTENCY-INDUCING SMALL COMPOUND (PLU) has also been recognized as a callus inducer, and its application induces callus formation through activating the auxin signaling cascade [23]. Subsequent investigations reveal that HEAT SHOCK PROTEIN 90 (HSP90) plays a crucial role in mediating early PLU responses, and PLU potentiates auxin signaling through the upregulation of auxin receptor gene expression in an HSP90-dependent manner (Fig. 1). In a related pathway, 4-phenylbutyric acid (4PBA) is metabolized into the natural auxin phenylacetic acid, which in turn promotes callus formation and subsequent shoot regeneration (Fig. 1; [25]). Notably, this regeneration-promoting role of 4PBA operates independently of its known activity as an HDAC inhibitor.

Metabolomic studies have revealed that the AMP accumulates within pluripotent callus [24]. Exogenous application of AMP selectively enhances the regeneration potential of hypocotyl- and root-derived explants, underscoring AMP as a pivotal metabolite in conferring regeneration competence during tissue culture [24]. RNA-Seq data show that AMP upregulates its own biosynthetic genes, such as *AMIDOPHOSPHORIBOSYLTRANSFERASE 1* (*ASE1*) and *ASE2*. In addition, AMP modulates the expression of *PLETHORA* (*PLT*) transcription factors [24], which are essential for callus pluripotency acquisition [26]. Inconsistent, the *plt3 plt5 plt7* triple mutant is insensitive to AMP, indicating that AMP promotes pluripotency in a PLT-dependent manner. Notably, AMP treatment strongly enhances callus formation and shoot regeneration in cabbage and tomato, as well as somatic embryogenesis in *Peucedanum japonicum*. Collectively, these findings establish AMP as a potent and broadly effective enhancer of plant regeneration across a wide range of plant species (Fig. 1; [24]).

The Phenyl-adenine (Phe-Ade, N phenyl-9H-purin-6-amine) has been identified as an inducer of shoot regeneration regulator LIGHT-DEPENDENT SHORT HYPOCOTYLS4 [27]. Treatment with Phe-Ade significantly promotes shoot regeneration in a dose-dependent manner. Subsequent assay shows that Phe-Ade provokes transcriptional levels of cytokinin responsive genes, including *ARABIDOPSIS RESPONSE REGULATOR 2* (*ARR2*), *ARR10*, and *ARR14* [27]. On the other hand, while Phe-Ade regulates both the expression and enzymatic activity of *CYTOKININ OXIDASE/DEHYDROGENASE* (*CKX*), it does not significantly affect the expression of cytokinin biosynthetic genes such as *ISOPENTENYL TRANSFERASEs* (*IPT*s), *CYP735A2*, and *LONELY GUY*s (*LOG*s). Further analysis indicates that Phe-Ade activates cytokinin signaling primarily through the receptors ARABIDOPSIS HISTIDINE KINASE3 (AHK3) and AHK4 [27]. Together, these findings support that Phe-Ade represents a cytokinin-like compound that induces shoot regeneration (Fig. 1).

The small molecule ZIC2 (3-chloro-N-(1,5-dimethyl-3-oxo-2-phenyl-2,3-dihydro-1H-pyrazol-4-yl)-1-benzothiophene-2-carboxamide) has been identified as a vital regulator that targets shoot regeneration-associated Class III homeodomain-leucine zipper (HD-ZIP III) transcription factors [28]. During shoot regeneration, treatment with ZIC2 significantly promotes cytokinin levels by increasing the expression of *IPT*s that are involved in cytokinin biosynthesis [28]. Concurrently, ZIC2 suppresses polar auxin transport by downregulating the expression of auxin *PIN-FORMED* (*PIN*) auxin efflux carriers [28]. The resultant reduction in auxin accumulation subsequently activates HD-ZIP III transcription factors, which in turn promote shoot regeneration (Fig. 1; [28]).

Trichostatin A (TSA), an HDACs inhibitor, enhances shoot regeneration in callus derived from root explants [29]. TSA application upregulates key regeneration-specific genes, including *WUS*, *RELATED TO AP2.6 L*, *SHOOT MERISTEMLESS* (*STM*), and *CUP-SHAPED COTYLEDON 2*. It also increases cytokinin sensitivity by enhancing the transcription levels of *ARR10* and *ARR12* as well as their downstream targets *ARR5*, *ARR7*, and *ARR15* [29]. This coordinated activation of cytokinin signaling thus facilitates shoot regeneration (Fig. 1).

### Bacterial cellulose induces plant tissue repair

Bacterial cellulose (BC), widely used in biomedical applications for human tissue regeneration, also promotes wound healing in plants [30]. When exogenously applied to damaged leaves, BC initiates the wound healing process, demonstrating its capacity in facilitating plant tissue repair. Further investigations have revealed two distinct mechanisms by which BC enhances plant regeneration. On one hand, BC application results in the accumulation of the phytohormones auxin and cytokinin, with cytokinin playing a pivotal role in BC-mediated regeneration. On the other hand, BC triggers defense signaling pathways that bolster plant regeneration, specifically by activating the defense-related transcription factor *WRKY8*. WRKY8 subsequently binds to the promoter region of *GLUTATHIONE TRANSFERASE phi7*, increasing its expression and leading to a sustained burst of reactive oxygen species (ROS). The continuous production and detoxification of ROS likely facilitate cellular proliferation, working in concert with cytokinin signaling to driving plant regeneration (Fig. 1; [30]).

### Glutathione regulates root regeneration via modulating cell cycle

Emerging evidence indicates that plant root regeneration necessitates coordinated cell division and identity transition in cells that will give rise to the regenerated tissue, a process in which glutathione (GSH) is implicated [31]. Single-cell RNA sequencing of regenerating versus nonregenerating cells following the excision of the root tip has demonstrated that regenerating cells predominantly accumulate during the G1-to-S phase transition, with a notable absence in G1 phase, suggesting a significant reduction in G1 duration during root regeneration [31]. Consistent with this, further investigation utilizing various marker lines reveals that cells within the regenerating meristem undergo an accelerated G1 phase. Additionally, staining with 7-amino-4-chloromethylcoumarin (Blue CMAC), a GSH-specific dye, showed a rapid increase in nuclear GSH across all root cell types after wounding, suggesting that cells undergo reprogramming first experience a localized burst of GSH influx into the nucleus, followed by synchronized G1 exit and rapid cell cycle progression. Supporting this, treatment with L-buthionine-sulfoximine, a GSH biosynthesis inhibitor, prolonged G1 phase and impaired root regeneration. Notably, exogenous GSH application enhanced regeneration efficiency after injury and symplastic blockage of ground tissue. Collectively, these findings indicate that ground tissue serves as the GSH source for facilitating G1 exit, abbreviating cell cycle, and rapid cellular reprogramming, thereby promoting root regeneration (Fig. 1).

### Small signaling peptides unlocks plant regeneration capacity

Small signaling peptides, encoded by nuclear genes, are secreted polypeptide-based intercellular communication signal molecules, which generally require proteolytic processing of preproproteins and post-translational modifications [32]. Routinely, small peptide molecules can be chemically synthesized by solid-phase peptide synthesis, representing attractive small molecules for critical applications. Similar to small molecules, synthetic peptides are exogenously applied in various bioassays in a controllable concentration- and time-dependent manner [32]. Many families of small signaling peptides, such as CLAVATA3 (CLV3)/EMBRYO SURROUNDING REGION-RELATED (CLE), PLANT ELICITOR PEPTIDE, C-TERMINALLY ENCODED PEPTIDE (CEP) and RAPID ALKALINIZATION FACTOR (RALF) families, are preferentially expressed during the distinct processes of callus formation and shoot regeneration, thereby emphasizing their essential roles during plant regeneration [33]. Exogenous application of chemically synthetic CLE1–7 or CLE9/10 peptides suppresses *de novo* shoot regeneration in a dose-dependent manner without affecting callus formation [34, 35]. Genetic studies indicate that *clv1* and *bam1* receptor mutants are insensitive to these CLE peptides, implicating that CLV1 and BAM1 as potential receptors for CLE-mediated *de novo* shoot regeneration [34, 35]. CLE1–7/9/10 peptides downregulate *WUS* expression, further explaining their negative effect on *de novo* shoot regeneration [34, 35]. Similarly, callus-expressed CLE16, CLE17, CLE19, CLE27, and CLE40 are negative regulators in shooting ability because their loss-of-function mutants exhibit significant promotion in shoot regeneration capacity [9]. To meet the practical desire to promote shoot regeneration, these regeneration-inhibitory CLE peptides were chosen to engineer into unnatural peptide variants that could bolster shoot regeneration [9]. Intriguingly, a glycine-to-threonine substitution in the CLE motif of CLE16, CLE17, CLE19, and CLE40 peptides created potent peptide variants that could markedly promote *de novo* shoot regeneration without adversely affecting plant vitality [9].

The tomato REGENERATION FACTOR1 (REF1) peptide is a systemin-independent local wound signal that could promote both callus formation and shoot regeneration in a dose-responsive manner [36]. REF1 binds to the PEPR1/2 ORTHOLOG RECEPTOR-LIKE KINASE1 (PORK1) receptor and subsequently elevate the transcriptional levels of *WOUND-INDUCED DEDIFFERENTIATION 1* (*WIND1*), a key regulator of wound-induced cellular reprogramming, eventually promoting plant regenerative capacity (Fig. 1; [36]). By contrast, simultaneous loss of callus-expressed *CEP13/14/15* genes resulted in an increased adventitious shoot capacity [37]. Thus, successful shoot regeneration involves robust coordinating positive regulators and negative regulators to ensure the accurate initiation, progression, and completion.

Following excision of the root apex, a new root apex regenerates within days through the coordinated activation and deactivation of multiple signaling pathways [38, 39]. Wounding triggers RALF33 accumulation and exogenous application of chemically synthetic RALF33 peptides increases root regeneration rates, whereas the *ralf33* mutant exhibits reduced regeneration capability [40]. Furthermore, synthetic RALF33 fails to enhance root regeneration in the *fer* mutant, confirming that the FERONIA (FER) receptor is required for RALF33-mediated root regeneration [40]. Mechanistically, elevated RALF33 peptides inhibit FER-mediated phosphorylation of TOPLESS-RELATED4 (TPR4), impairing its interaction with the transcription factor ETHYLENE RESPONSE FACTOR115 (ERF115), a master regulator in root-tip regeneration [40, 41]). The disassociation of TPR4 with ERF115 releases the transcriptional activity of *ERF115*, thereby promoting root regeneration rates (Fig. 1; [40]).

Phytosulfokine (PSK) is a tyrosine-sulfated pentapeptide widely distributed across the plant kingdom and plays critical roles in regulating plant growth, development, and responses to biotic and abiotic stresses. In *Cunninghamia lanceolata*, application of synthetic PSK peptides markedly reduces browning in proembryogenic masses and enhances the formation of proembryos [42], underscoring its importance in somatic embryogenesis. Transcriptomic analyses further reveal that treatment with PSK peptides results in the downregulation of enzymes involved in H_2_O_2_ biosynthesis. The reduction in H_2_O_2_ levels subsequently activates the expression of somatic embryogenesis-related genes such as *WOX2*, *WUS*, and *CLV3*, thereby promoting somatic embryogenesis (Fig. 1).

### Concluding remarks and future perspectives

Genetic transformation and genome editing are crucial for studying gene functions and improving crop varieties. However, low plant regeneration efficiencies and genotype-dependent regeneration heavily restrict plant transformation and genome editing for crop enhancement. Conventional techniques to overcome the low regeneration capacity are often limited by technical complexities and genotype-dependent regeneration [43]. Through innovating the formulation of regeneration media by combinations of specific chemicals, small molecules offer an effective, inexpensive, and convenient strategy to tackle these challenges by enhancing regeneration efficiency in a stepwise and controllable manner. Furthermore, small molecules possess beneficial properties, such as non-integration into the genome and easy manufacturing and standardization, thereby holding significant promise to precise control of plant regeneration in a highly controlled manner with desired dose and time. While numerous small molecules capable of inducing pluripotency have been identified in mammalian systems [10–12], their potential to modulate plant regeneration remains largely unexplored and thus necessitates further investigations. Given the similarities shared in regeneration of plants and animals [44–46]), it is tempting to envisage the application of small molecules identified from mammals in plant regeneration. This further highlights the critical need to systematic evaluation of mammalian-derived small molecules for their ability to improve plant regeneration and transformation. Indeed, a number of mammalian-derived small molecules has been shown as powerful tools to significantly enhance plant regeneration capacity (Fig. 1). The demonstrated potency of small molecules in plant regeneration emphasizes the importance of discovering new small compounds and engineering them into more effective derivatives.

**Figure 2:**
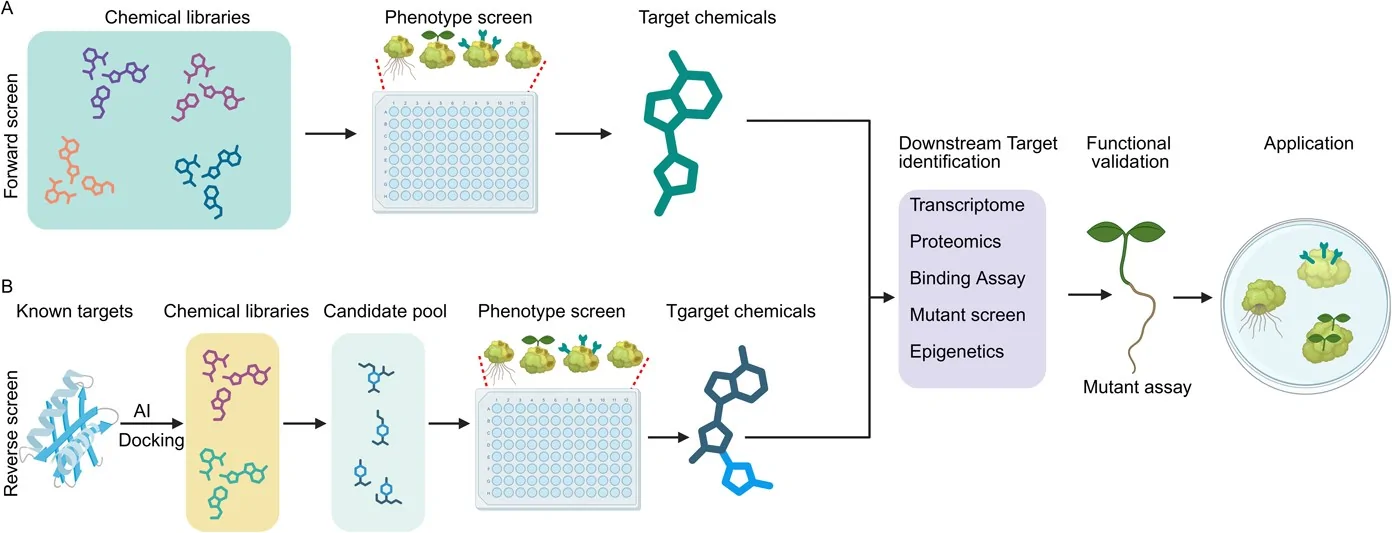
Approaches to identify small chemical molecules and their targets that can improve plant regeneration efficiency. (A) A forward chemical screening (phenotype-driven) involves the evaluation of compound libraries to identify specific phenotypic responses, followed by the elucidation of target proteins through binding assays, transcriptomic and epigenomic analyses, proteomic profiling, as well as the examination of mutant plants. (B) A reverse chemical screening approach (target-centric) utilizing protein structure-based assays followed by docking simulations to identify target compounds. The isolated small molecules may then be evaluated for specific phenotypic effects. Various methodologies, including binding assays, transcriptomic and epigenomic analyses, and proteomics, could be additionally employed to elucidate potential targets, thus facilitating their application in crop improvements.

In plant biology, numerous genes involved in regeneration have been functionally characterized, particularly key pluripotency factors such as WUS, STM, and PLTs [47, 48] ). Currently, most small molecules used in plant regeneration function mainly to activate pluripotency factors or to reshape the epigenetic landscape by erasing somatic cell identities. As such, to identify more novel small molecules that facilitate plant regeneration, an integrated strategy combining forward (phenotype-driven) and reverse (target-driven) chemical screens with physiological, biochemical, and computational approaches is needed to be implemented (Fig. 2).

Chemical libraries such as the ZINC library (http://zinc.docking.org) and the ChemBridge Compound Library (https://chembidge.com/screening-compounds/lead-like-druglike-compounds) provide valuable resources for screening of small molecules that may selectively target transcription factors and modulate their activity in the context of plant regeneration[47, 48] . In parallel, innovations in chemical screening, such as refinements in using virtual libraries, will allow for the rapid creation of effective small molecules. A further rational design of these small molecules is expected to improve their superiorities. Moreover, engineering native small signaling peptides through amino acid substitution or chemical modification represents a promising strategy to enhance their efficacy in plant regeneration [49]. The next steps toward practical application involves determining the proper concentration and treatment time and *de novo* designing and synthesizing of chemical cocktails. Nevertheless, future investigations need to elucidate the underlying mechanisms by which small molecules impact plant regeneration processes, with particular regard to chemical reprogramming cell fates and exploring their targets.

In conclusion, chemical reprogramming using small molecules offers a targeted strategy to induce plant pluripotency. Leveraging artificial intelligence tools with multi-omics data enables efficient screening and prediction of desired small molecules, facilitating the identification of a set of compounds that enhance regeneration plasticity. Understanding of small molecule-mediated cellular reprogramming will unveil new principles of cell fate regulation, thus offering a fresh perspective on plant cell plasticity and regenerative potential. Practically, the strategy of small molecule-regulated plant regeneration holds significant potential to advance crop improvement, ultimately leading to enhanced crop productivity and resilience.
